# Altered Hepatic Hydrogen Sulfide-Related Homeostasis in Metabolic Dysfunction-Associated Steatotic Liver Disease and Its Changes Following Sleeve Gastrectomy

**DOI:** 10.3390/jcm15218514

**Published:** 2026-11-02

**Authors:** Yitong Han, Junhang Chen, Yi Xu, Longhao Sun, Xiangchen Dai

**Affiliations:** 1 Department of General Surgery, Tianjin Medical University General Hospital, Tianjin 300052, China; hanhytlcyx@163.com (Y.H.);; 2 Tianjin Key Laboratory of Precise Vascular Reconstruction and Organ Function Repair, Tianjin 300052, China; 3 Tianjin General Surgery Institute, Tianjin 300052, China; 4 Department of Vascular Surgery, Tianjin Union Medical Center, The First Affiliated Hospital of Nankai University, Tianjin 300121, China

**Keywords:** metabolic dysfunction-associated steatotic liver disease, hydrogen sulfide, sleeve gastrectomy, obesity

## Abstract

**Background:** Metabolic dysfunction-associated steatotic liver disease (MASLD) is closely associated with obesity and metabolic dysfunction. Hydrogen sulfide (H_2_S) is involved in hepatic glucose and lipid metabolism and redox homeostasis, but alterations in hepatic H_2_S-related homeostasis in MASLD and their changes following sleeve gastrectomy (SG) remain incompletely understood. This study investigated hepatic H_2_S metabolism in MASLD and its changes following SG. **Methods:** Liver tissues from five patients with MASLD and five non-MASLD controls were analyzed for histology, H_2_S levels, and expression of cystathionine β-synthase (CBS), cystathionine γ-lyase (CSE), and 3-mercaptopyruvate sulfurtransferase (MPST) by qPCR, Western blotting, and immunohistochemistry. A high-fat diet-induced obese rat model was used to evaluate the metabolic, biochemical, histological, and hepatic H_2_S-related effects of SG. **Results:** Patients with MASLD showed marked hepatic steatosis, a reduced hepatic H_2_S-related sulfide pool and CSE expression, increased MPST expression, and unchanged CBS expression. Similar alterations were observed in obese rats. Compared with Sham rats, SG reduced body weight and food intake, improved glucose and lipid metabolism and liver function, and attenuated hepatic steatosis. These improvements were accompanied by an increased hepatic H_2_S-related sulfide pool and CSE expression and decreased MPST expression, whereas CBS remained stable. **Conclusions:** MASLD was associated with altered hepatic H_2_S-related homeostasis characterized by a reduced H_2_S-related sulfide pool, decreased CSE expression, increased MPST expression, and relatively stable CBS expression. In obese rats, metabolic improvement following SG was accompanied by corresponding changes in hepatic H_2_S-related homeostasis toward the pattern observed in controls. Whether these H_2_S-related changes contribute functionally to the metabolic benefits of SG remains to be determined.

## 1. Introduction

Metabolic dysfunction-associated steatotic liver disease (MASLD) is one of the most common chronic liver diseases worldwide, encompassing a spectrum ranging from simple hepatic steatosis to metabolic dysfunction-associated steatohepatitis (MASH), liver fibrosis, and ultimately cirrhosis. In recent years, the global burden of MASLD has increased substantially in parallel with the rising prevalence of obesity, insulin resistance, and other metabolic disorders. Large-scale systematic reviews have estimated that the global prevalence of MASLD among adults is approaching or exceeding 30%, with an even higher prevalence among individuals with overweight or obesity, highlighting the close relationship between MASLD and obesity-associated metabolic disturbances [1,2]. Beyond its hepatic manifestations, MASLD is increasingly recognized as a systemic metabolic disorder associated with clinically relevant extrahepatic complications, including cardiovascular and chronic kidney disease [3]. Therefore, elucidating the metabolic mechanisms underlying obesity-associated MASLD and identifying effective interventions to ameliorate hepatic steatosis and metabolic injury remain clinically important.

Weight loss is a key therapeutic strategy for obesity-associated MASLD. In patients with severe obesity, metabolic and bariatric surgery can achieve sustained weight reduction and improve glucose and lipid metabolism, insulin resistance, and multiple obesity-related comorbidities. Previous systematic reviews and meta-analyses have shown that bariatric surgery can markedly improve hepatic steatosis, inflammation, and, in some patients, fibrosis [4,5]. Sleeve gastrectomy (SG), one of the most widely performed bariatric procedures, exerts metabolic benefits that cannot be fully explained by gastric restriction and reduced caloric intake alone. Emerging evidence suggests that alterations in bile acid signaling, gut hormones, insulin sensitivity, lipid metabolism, systemic inflammation, and the gut microbiota may all contribute to the metabolic effects of SG [6]. However, the hepatic metabolic mechanisms underlying the improvement of fatty liver disease after SG remain incompletely understood, and identification of additional metabolic signaling pathways associated with postoperative hepatic improvement is warranted.

Hydrogen sulfide (H_2_S) is an important endogenous gaseous signaling molecule involved in hepatic glucose and lipid metabolism, redox homeostasis, inflammatory responses, and cellular stress regulation. Endogenous H_2_S in mammals is generated mainly through enzymatic pathways involving cystathionine β-synthase (CBS), cystathionine γ-lyase (CSE), and 3-mercaptopyruvate sulfurtransferase (MPST), all of which are expressed in the liver [7]. Previous experimental studies have shown that high-fat feeding reduces hepatic H_2_S-generating capacity and is accompanied by decreased hepatic CSE expression [8], whereas exogenous H_2_S supplementation can attenuate high-fat diet-induced hepatic lipid accumulation and metabolic injury [9]. Further genetic evidence has demonstrated that hepatocyte-specific CSE deficiency aggravates high-fat diet-induced hepatic lipid accumulation, insulin resistance, and related metabolic abnormalities, whereas restoration of H_2_S signaling partially reverses these phenotypes, suggesting that the hepatocellular CSE/H_2_S axis may represent an important regulatory system for maintaining hepatic metabolic homeostasis [10]. However, whether SG is accompanied by alterations in hepatic H_2_S-related homeostasis and whether postoperative changes in H_2_S are accompanied by remodeling of the expression profiles of CSE, CBS, and MPST remain insufficiently characterized. Therefore, the present study aimed to characterize hepatic H_2_S-related alterations in MASLD and to determine whether these alterations were accompanied by corresponding changes after SG in obese rats.

## 2. Methods

### 2.1. Animals

Male Sprague–Dawley rats aged 8–10 weeks and weighing 300–350 g were purchased from Beijing SPF Biotechnology Co., Ltd. (Beijing, China). Animals were housed under standard conditions (20–25 °C, 40–60% humidity, 12-h light/dark cycle) with free access to food and water unless otherwise specified. Standard chow and high-fat diet (HFD) were obtained from Beijing HFK Bioscience Co., Ltd. (Beijing, China). The HFD consisted of H10060 supplemented with 2.5% cholesterol. Eight rats were included in each experimental group. No formal a priori sample-size calculation was performed; the group size was determined based on sample sizes used in comparable experimental studies and experimental feasibility. Rats were randomly allocated to the experimental groups before the corresponding dietary or surgical interventions. The study is reported in accordance with the ARRIVE guidelines, and the completed ARRIVE checklist is provided as Supplementary Material (File S1).

### 2.2. High-Fat Diet-Induced Obesity and Fatty Liver Model

After 1 week of acclimatization on standard chow, rats were randomly assigned to the Control group or HFD group. Control rats remained on standard chow, whereas HFD rats were fed the HFD for 12 weeks. Body weight and food intake were monitored regularly. After 12 weeks, one HFD-fed rat was randomly euthanized for liver histology, and successful model induction was confirmed by marked hepatic steatosis. The remaining HFD-fed rats were then randomly assigned to the Sham or sleeve gastrectomy (SG) group.

### 2.3. Sleeve Gastrectomy and Sham Surgery

Rats were fasted for 12 h and deprived of water for 8 h before surgery. General anesthesia was induced and maintained with inhaled isoflurane. After abdominal preparation under aseptic conditions, a 2–3 cm midline incision was made below the xiphoid process. The stomach was exposed, and the perigastric ligaments and connective tissues were dissected to mobilize the fundus and greater curvature. After ligation of the relevant perigastric vessels, approximately 70–80% of the stomach, including the fundus and most of the gastric body along the greater curvature, was resected. The remaining stomach was closed continuously with 4-0 absorbable sutures. After confirming the absence of bleeding, leakage, or stenosis, the stomach was returned to the abdominal cavity, and the abdominal wall was closed in layers. Sham rats underwent the same preoperative preparation, isoflurane anesthesia, abdominal incision, and perigastric dissection as SG rats. A small portion of the gastric wall along the greater curvature was resected and sutured to mimic surgical injury without extensive gastric resection. The operative duration was kept similar to that of SG.

### 2.4. Postoperative Care and Follow-Up

Postoperatively, rats were closely monitored and received 5 mL of 5% glucose–saline solution subcutaneously once daily for 3 days. Food and water were withheld on postoperative day 1; limited water was provided on day 2, free water on day 3, liquid diet on day 4, and solid food from day 6 onward.

After resumption of solid feeding, Sham and SG rats continued the same HFD, whereas Control rats remained on standard chow. Animals were followed for 8 weeks, with regular monitoring of body weight, food intake, and fasting blood glucose. At the end of follow-up, rats were euthanized by isoflurane overdose, and blood and liver tissues were collected for biochemical, histological, H_2_S, qPCR, Western blot, and immunohistochemical analyses.

### 2.5. Human

A total of 10 participants undergoing surgery in the Department of General Surgery at Tianjin Medical University General Hospital were included, comprising five patients with MASLD and five non-MASLD controls. Liver tissues from the MASLD group were obtained from patients with obesity undergoing metabolic and bariatric surgery. Control liver tissues were obtained from patients undergoing surgical resection for hepatic hemangioma, and non-lesional liver tissue adjacent to the hemangioma was collected. MASLD was assessed based on clinical information and histopathological findings. Patients with viral hepatitis, alcohol-related liver disease, autoimmune liver disease, drug-induced liver injury, or other chronic liver diseases of known etiology were excluded. In the Control group, non-lesional liver tissues were histologically confirmed to have no apparent hepatic steatosis, and patients with other chronic liver diseases or severe metabolic disorders were excluded. Demographic and perioperative laboratory data, including age, height, body weight, body mass index (BMI), liver function tests, fasting blood glucose, and lipid profiles, were collected. Fresh liver tissues were immediately divided after surgical collection. A portion of each sample was fixed in 4% paraformaldehyde for histological staining, whereas the remaining tissue was rapidly frozen and stored at −80 °C for measurement of hepatic H_2_S levels, quantitative real-time PCR, and Western blot analysis.

### 2.6. Biochemical Analysis

Blood samples were collected after an overnight fast and centrifuged to obtain serum for biochemical analyses. Serum levels of alanine aminotransferase (ALT), aspartate aminotransferase (AST), total cholesterol (TC), triglycerides (TG), high-density lipoprotein cholesterol (HDL-C), and low-density lipoprotein cholesterol (LDL-C) were measured using commercially available assay kits (Nanjing Jiancheng Bioengineering Institute, Nanjing, China) according to the manufacturer’s instructions. Serum inflammatory cytokines, including interleukin-1β (IL-1β), interleukin-6 (IL-6), and tumor necrosis factor-α (TNF-α), were measured using enzyme-linked immunosorbent assay kits (MultiSciences Biotech Co., Ltd., Hangzhou, China) according to the manufacturer’s protocols.

### 2.7. Hepatic H_2_S-Related Sulfide Pool

The hepatic H_2_S-related sulfide pool was assessed using a commercial fluorescence-based assay kit (BC205F; Solarbio Science, Beijing, China) according to the manufacturer’s instructions. Briefly, frozen liver tissues were weighed, homogenized on ice in extraction buffer, and centrifuged at 10,000× *g* for 10 min at 4 °C. The resulting supernatant was kept on ice and used for fluorescence-based sulfide detection. A fluorescence microplate reader was preheated for 30 min, and fluorescence was measured at an excitation wavelength of 355 nm and an emission wavelength of 445 nm, with distilled water used as the blank. Fluorescence-derived values were calculated from the standard curve, normalized to tissue weight, and expressed as nmol/g tissue. Because this fluorescence-based assay does not directly measure the instantaneous concentration of free H_2_S or the rate of enzymatic H_2_S production, the measured values were interpreted as reflecting the detectable hepatic H_2_S-related sulfide pool under the assay conditions. Further procedural details followed the manufacturer’s protocol.

### 2.8. Histological Examination

Liver samples were fixed in 4% paraformaldehyde (PFA) for histological examination. Paraffin-embedded liver tissues were sectioned at a thickness of 5 μm, deparaffinized, rehydrated, and stained with hematoxylin and eosin (H&E) or Masson’s trichrome staining kits (G1125 and G1340; Solarbio Science, Beijing, China) according to the manufacturer’s instructions. For Oil Red O staining, liver tissues were fixed in 4% PFA, embedded in optimal cutting temperature (OCT) compound, and sectioned at a thickness of 7 μm using a cryostat. Frozen sections were stained using an Oil Red O staining kit (G1261; Solarbio Science, Beijing, China) according to the manufacturer’s protocol. Histological assessment was performed by an experienced hepatologist who was blinded to group allocation to minimize assessment bias. Hepatic histological activity was evaluated using the NAFLD Activity Score (NAS). The NAS was calculated as the unweighted sum of three histological components: steatosis (0–3), lobular inflammation (0–3), and hepatocellular ballooning (0–2), yielding a total score ranging from 0 to 8. Steatosis was scored as 0 (<5%), 1 (5–33%), 2 (>33–66%), or 3 (>66%) according to the proportion of affected hepatocytes. Lobular inflammation was scored as 0 (no foci), 1 (<2 foci per ×200 field), 2 (2–4 foci per ×200 field), or 3 (>4 foci per ×200 field). Hepatocellular ballooning was scored as 0 (none), 1 (few ballooned hepatocytes), or 2 (many cells/prominent ballooning). Higher NAS values indicate greater histological disease activity. NAS was used as a semiquantitative measure of histological activity rather than as a standalone diagnostic criterion for MASH [11,12].

### 2.9. Immunohistochemical (IHC) Staining

Paraffin-embedded liver sections (5 μm) were deparaffinized and rehydrated. Antigen retrieval was performed by heating the sections in sodium citrate antigen retrieval solution (C1032; Solarbio Science, Beijing, China) for 20 min. After washing with phosphate-buffered saline (PBS), endogenous peroxidase activity was blocked with hydrogen peroxide, followed by blocking with goat serum (C0265; Beyotime Biotechnology, Shanghai, China) at room temperature.

The sections were then incubated overnight at 4 °C with primary antibodies against cystathionine β-synthase (CBS; 14787-1-AP; Proteintech Group, Wuhan, China), cystathionine γ-lyase (CSE; 12217-1-AP; Proteintech Group, Wuhan, China), and 3-mercaptopyruvate sulfurtransferase (MPST; sc-376168; Santa Cruz Biotechnology, Dallas, TX, USA). After washing with PBS, the sections were incubated with a biotin-conjugated secondary antibody (RGAR011; Proteintech Group, Wuhan, China) for 30 min at room temperature. Immunoreactivity was visualized using 3,3′-diaminobenzidine (DAB; ZLI-9018; ZSGB-BIO, Beijing, China), followed by hematoxylin counterstaining. Finally, the sections were dehydrated, mounted, and allowed to dry before microscopic evaluation.

### 2.10. Quantitative Reverse Transcription-Polymerase Chain Reaction (qRT-PCR)

Total RNA was extracted from liver tissues using Trizol reagent followed by magnetic bead-based purification. RNA quality was assessed to ensure its suitability for downstream analysis. Purified total RNA was reverse-transcribed into first-strand cDNA using a reverse transcription kit (R323-01; Vazyme Biotech Co., Ltd., Nanjing, China). Quantitative real-time PCR (RT-qPCR) was performed using Taq Pro Universal SYBR qPCR Master Mix (Q712-02; Vazyme Biotech Co., Ltd., Nanjing, China) according to the manufacturer’s instructions. Relative gene expression was calculated using the 2^−ΔΔCt^ method. The primer sequences used in this study are listed in Table 1.

### 2.11. Western Blot Analysis

Liver tissues were homogenized in RIPA lysis buffer using a bead-based homogenizer and centrifuged at 12,000× *g* for 10 min at 4 °C. Protein concentrations were determined using a BCA protein assay kit (PC0020; Solarbio Science, Beijing, China). Equal amounts of protein (20 μg) were separated by 10% SDS–PAGE and transferred onto 0.45-μm polyvinylidene difluoride (PVDF) membranes (Merck Millipore, Darmstadt, Germany). The membranes were blocked with 5% non-fat milk in Tris-buffered saline containing Tween-20 (TBST) for 2 h at room temperature and then incubated overnight at 4 °C with primary antibodies against CBS (14787-1-AP; Proteintech Group, Wuhan, China), CSE (12217-1-AP; Proteintech Group), MPST (sc-376168; Santa Cruz Biotechnology, Dallas, TX, USA), and β-actin (20536-1-AP; Proteintech Group). After washing with TBST, the membranes were incubated for 1 h at room temperature with horseradish peroxidase-conjugated goat anti-rabbit (SA00001-2; Proteintech Group) or goat anti-mouse (SA00001-1; Proteintech Group) secondary antibodies, as appropriate. Protein bands were visualized using an enhanced chemiluminescence detection system.

### 2.12. Statistical Analysis

Statistical analyses were performed using IBM SPSS Statistics version 22.0, and graphical presentation was performed using GraphPad Prism version 9.5. Continuous variables were analyzed according to their distributional characteristics. Normally distributed continuous variables are presented as the mean ± standard deviation (SD), and comparisons between two independent groups were performed using an unpaired two-tailed Student’s *t* test. Non-normally distributed continuous variables are presented as the median (interquartile range, IQR) and were compared using the Mann–Whitney U test. Categorical variables are presented as counts or percentages and were compared using Fisher’s exact test, as appropriate. Comparisons among the Control, Sham, and SG groups for endpoint continuous variables were performed using ordinary one-way analysis of variance (ANOVA), followed by Tukey’s multiple-comparison test for pairwise comparisons. All statistical tests were two-sided, and a *p* value < 0.05 was considered statistically significant.

## 3. Results

### 3.1. Clinical Characteristics and Alterations in Hepatic H_2_S-Related Homeostasis in Patients with MASLD

**Figure 1:**
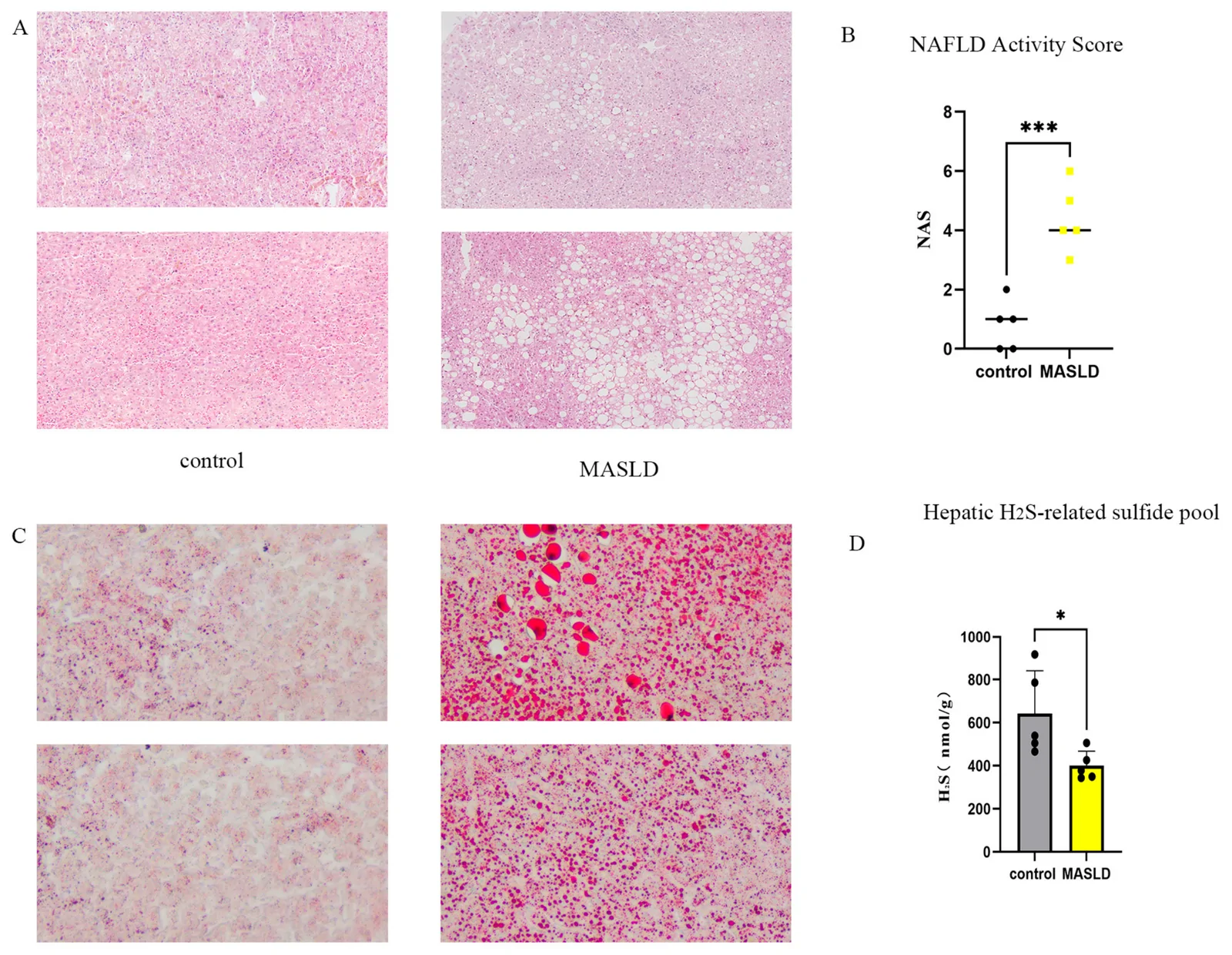
Hepatic steatosis is accompanied by a reduced hepatic H_2_S-related sulfide pool in patients with MASLD. (**A**) Representative hematoxylin and eosin (H&E)-stained liver sections from non-MASLD controls and patients with MASLD. (**B**) NAFLD Activity Score (NAS) in the Control and MASLD groups. (**C**) Representative Oil Red O-stained liver sections showing hepatic lipid accumulation in the two groups. (**D**) Hepatic H_2_S-related sulfide pool in the Control and MASLD groups. Compared with controls, patients with MASLD exhibited more pronounced hepatic steatosis, higher NAS, and a lower hepatic H_2_S-related sulfide pool. Data are presented as mean ± SD where applicable. * *p* < 0.05 and *** *p* < 0.001.

A total of 10 participants were included in the clinical analysis, comprising five patients with MASLD and five non-MASLD controls. The baseline clinical and biochemical characteristics of the participants are summarized in Table 2. No significant differences were observed between the two groups in age, height, total bilirubin (TBil), or direct bilirubin (DBil) (all *p* > 0.05). Compared with the Control group, patients with MASLD had a significantly higher BMI (*p* = 0.008), ALT (*p* = 0.001), AST (*p* = 0.009), TC (*p* = 0.009), TG (*p* = 0.033), and LDL-C (*p* < 0.001), together with significantly lower ALB (*p* = 0.034) and HDL-C (*p* = 0.002). Fasting glucose levels also differed significantly between the groups (*p* = 0.019). These findings demonstrate marked differences in metabolic and hepatic biochemical profiles between the two groups. We next examined hepatic histopathology and the H_2_S-related sulfide pool in the two groups. H&E staining showed largely preserved hepatic architecture in the Control group, whereas liver tissues from patients with MASLD exhibited prominent hepatocellular steatosis and lipid vacuolation. Consistently, Oil Red O staining revealed markedly increased lipid accumulation in the MASLD group, confirming substantial hepatic steatosis (Figure 1A–C). Fluorescence-based analysis showed a significantly lower hepatic H_2_S-related sulfide pool in patients with MASLD than in non-MASLD surgical controls (Figure 1D), indicating a reduction in the detectable hepatic H_2_S-related sulfide pool.

**Figure 2:**
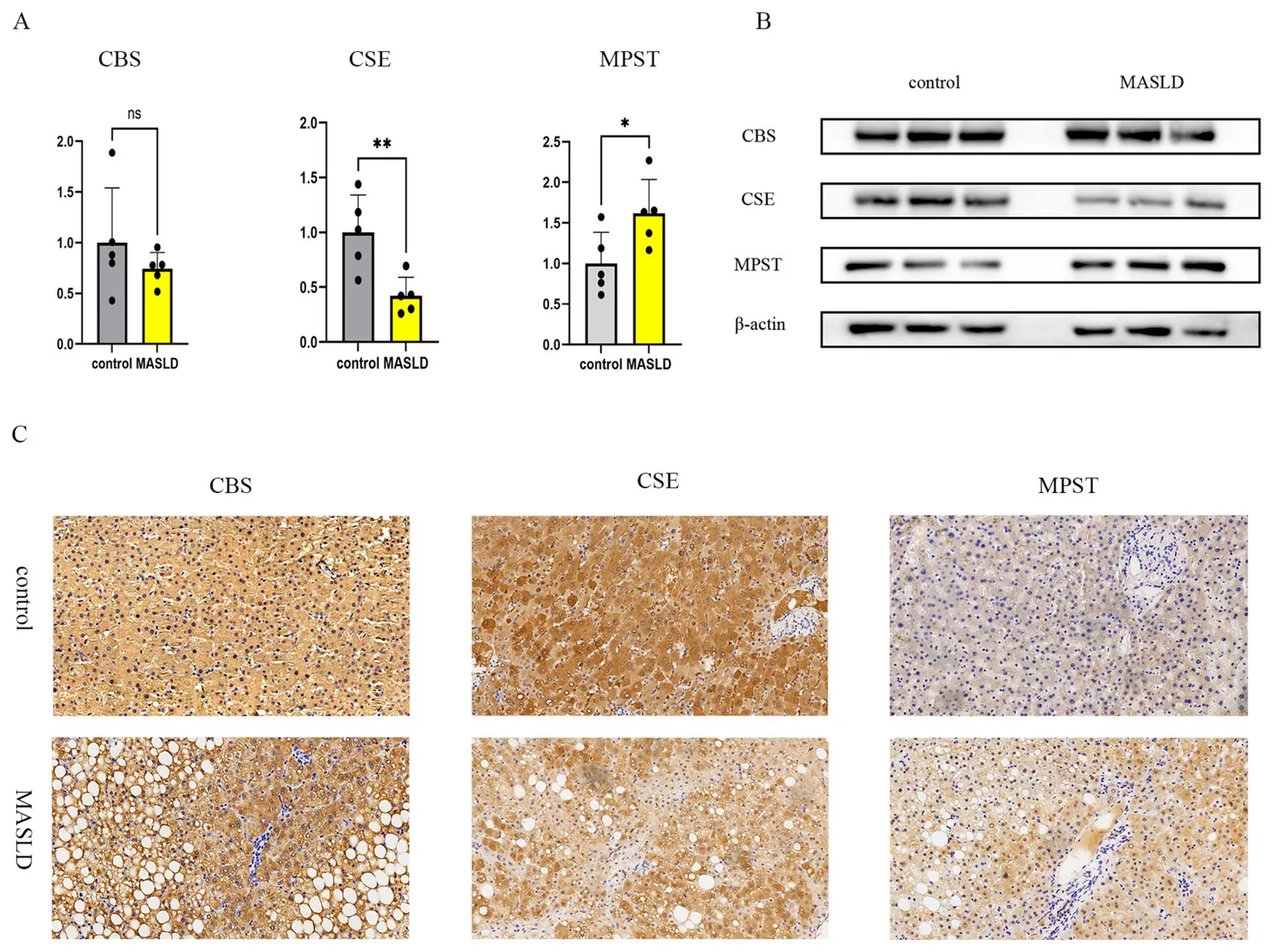
Altered hepatic expression of H_2_S-generating enzymes in patients with MASLD. Expression of cystathionine β-synthase (CBS), cystathionine γ-lyase (CSE), and 3-mercaptopyruvate sulfurtransferase (MPST) in liver tissues from non-MASLD controls and patients with MASLD. (**A**) Relative mRNA expression of CBS, CSE, and MPST determined by quantitative real-time PCR. (**B**) Representative Western blot images of CBS, CSE, and MPST, with β-actin used as the loading control. (**C**) Representative immunohistochemical staining of CBS, CSE, and MPST in liver tissues. CSE expression was lower and MPST expression was higher in the MASLD group than in controls, whereas CBS expression did not differ significantly between the groups. Data are presented as mean ± SD. * *p* < 0.05, ** *p* < 0.01; ns, not significant.

To determine whether the lower hepatic H_2_S-related sulfide pool was accompanied by changes in the expression of H_2_S-generating enzymes, we examined cystathionine β-synthase (CBS), cystathionine γ-lyase (CSE), and 3-mercaptopyruvate sulfurtransferase (MPST). qPCR analysis showed that CSE mRNA expression was significantly decreased, whereas MPST mRNA expression was significantly increased, in MASLD liver tissues compared with controls. In contrast, CBS mRNA expression did not differ significantly between the two groups (Figure 2A). Western blot analysis further confirmed a marked reduction in CSE protein expression and an increase in MPST protein expression in the MASLD group, while CBS protein levels remained unchanged (Figure 2B). Immunohistochemical staining showed an overall expression pattern consistent with the qPCR and Western blot findings (Figure 2C).

Collectively, these findings indicate that MASLD is associated with a lower hepatic H_2_S-related sulfide pool together with a distinct H_2_S-generating enzyme expression pattern characterized by decreased CSE expression, increased MPST expression, and relatively unchanged CBS expression.

### 3.2. Sleeve Gastrectomy Improves Body Weight and Metabolic Abnormalities in High-Fat Diet-Induced Obese Rats

**Figure 3:**
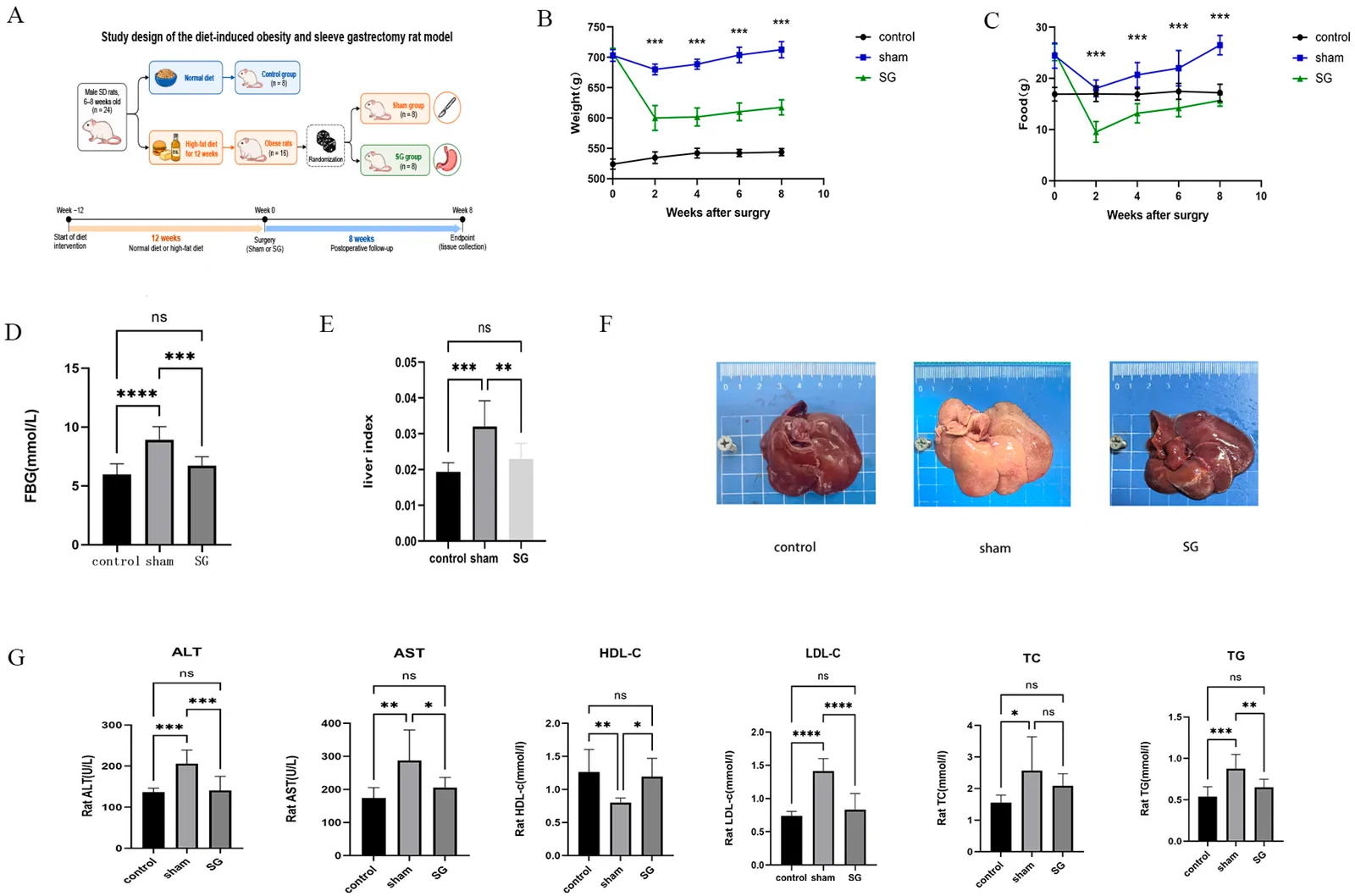
Sleeve gastrectomy ameliorates obesity-related metabolic abnormalities in high-fat diet-fed rats. (**A**) Schematic overview of the experimental design. Rats were fed either a normal diet or a high-fat diet (HFD) for 12 weeks. HFD-fed obese rats were subsequently randomized to sham surgery or sleeve gastrectomy (SG), followed by an 8-week postoperative period. (**B**) Changes in body weight during the postoperative follow-up. (**C**) Changes in food intake after surgery. (**D**) Fasting blood glucose (FBG) levels at the end of follow-up. (**E**) Liver index. (**F**) Representative gross appearance of livers from the Control, Sham, and SG groups. (**G**) Serum levels of alanine aminotransferase (ALT), aspartate aminotransferase (AST), high-density lipoprotein cholesterol (HDL-C), low-density lipoprotein cholesterol (LDL-C), total cholesterol (TC), and triglycerides (TG). Compared with Sham rats, SG reduced body weight and food intake and improved glucose metabolism, liver index, liver function, and most lipid parameters. For body weight and food intake, statistical significance indicates comparisons between the Sham and SG groups at each time point. Data are presented as mean ± SD. * *p* < 0.05, ** *p* < 0.01, *** *p* < 0.001, and **** *p* < 0.0001; ns, not significant.

To evaluate the effects of sleeve gastrectomy (SG) on obesity-related metabolic abnormalities, obesity was induced in rats by 12 weeks of high-fat diet feeding. Obese rats were subsequently randomized to undergo sham surgery or SG, while rats fed a normal diet served as the Control group. Animals were followed for 8 weeks after surgery (Figure 3A).

Body weight monitoring showed that rats in the Sham group maintained a persistently higher body weight throughout the postoperative period, whereas SG resulted in a rapid reduction in body weight, which remained significantly lower than that in the Sham group during the subsequent 8-week follow-up (Figure 3B). Food intake was also markedly reduced after SG. Although food intake gradually increased during postoperative recovery, it remained lower in the SG group than in the Sham group at the assessed time points (Figure 3C).

At 8 weeks after surgery, fasting blood glucose levels were significantly higher in the Sham group than in the Control group. SG significantly reduced fasting blood glucose compared with the Sham group, with no significant difference between the SG and Control groups (Figure 3D). Similarly, the liver index was significantly increased in the Sham group compared with the Control group, whereas SG significantly reduced the liver index to a level not significantly different from that of the Control group (Figure 3E). Gross examination of the liver showed a paler appearance and fatty liver-like morphological changes in the Sham group, whereas liver appearance was visibly improved after SG and more closely resembled that of the Control group (Figure 3F).

Serum biochemical analyses further demonstrated significant elevations in alanine aminotransferase (ALT) and aspartate aminotransferase (AST) levels in the Sham group compared with the Control group, consistent with obesity-associated liver injury. Both ALT and AST levels were significantly reduced after SG and did not differ significantly from those in the Control group (Figure 3G). With respect to lipid metabolism, the Sham group showed significantly decreased HDL-C levels and increased LDL-C, total cholesterol (TC), and triglyceride (TG) levels. SG significantly increased HDL-C and reduced LDL-C and TG levels, with no significant differences in these parameters between the SG and Control groups. Although TC levels tended to decrease after SG, the difference between the SG and Sham groups did not reach statistical significance (Figure 3G).

Overall, SG markedly reduced body weight and food intake, improved fasting blood glucose and liver index, ameliorated abnormalities in liver enzymes, and partially corrected obesity-associated dyslipidemia in high-fat diet-induced obese rats.

### 3.3. Sleeve Gastrectomy Alleviates Hepatic Steatosis and Histopathological Injury in Obese Rats

To assess the effects of SG on obesity-associated hepatic pathological changes, liver tissues from the three groups were subjected to histological analysis.

**Figure 4:**
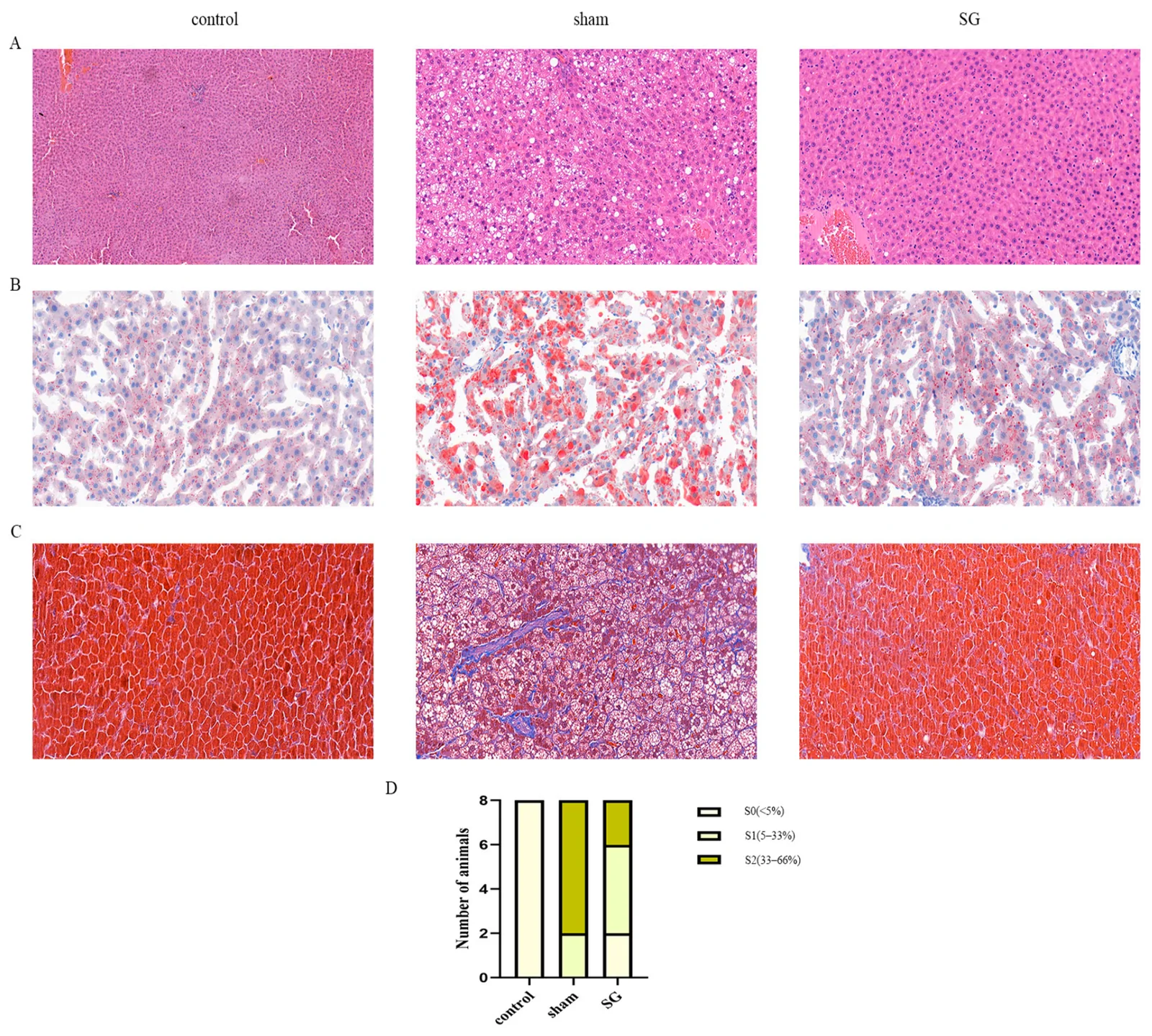
Representative histological changes in liver tissues from the Control, Sham, and SG groups. (**A**) H&E staining showing hepatic morphology and steatotic changes. (**B**) Oil Red O staining showing hepatic lipid accumulation. (**C**) Masson’s trichrome staining of liver tissues. (**D**) Distribution of hepatic steatosis grades among the three groups. Hepatic steatosis was classified according to the proportion of affected hepatocytes as S0 (<5%), S1 (5–33%), S2 (33–66%), and S3 (>66%). Compared with the Sham group, the SG group showed reduced hepatic lipid accumulation and a shift in the distribution of steatosis grades toward milder categories.

H&E staining showed preserved lobular architecture and regularly arranged hepatocytes in the Control group. In contrast, the Sham group exhibited prominent hepatocellular lipid vacuolation and steatosis, whereas these pathological alterations were markedly attenuated in the SG group (Figure 4A). Oil Red O staining demonstrated extensive lipid accumulation in the livers of Sham-operated rats, while hepatic lipid deposition was visibly reduced after SG (Figure 4B).

Additional histological staining revealed varying degrees of structural abnormalities and collagen deposition in the Sham group, which were attenuated after SG (Figure 4C). Consistently, histological grading of hepatic steatosis showed a higher proportion of animals with moderate steatosis in the Sham group, whereas the distribution of steatosis grades shifted toward milder categories following SG (Figure 4D).

These findings indicate that SG markedly attenuates high-fat diet-induced hepatic lipid accumulation and histopathological injury.

### 3.4. Changes in the Hepatic H_2_S-Related Sulfide Pool and Enzyme Expression Following Sleeve Gastrectomy in Obese Rats

Given the reduction in hepatic H_2_S levels and altered expression of H_2_S-producing enzymes observed in patients with MASLD, we next assessed these parameters in obese rats and compared the findings among the Control, Sham, and SG groups.

**Figure 5:**
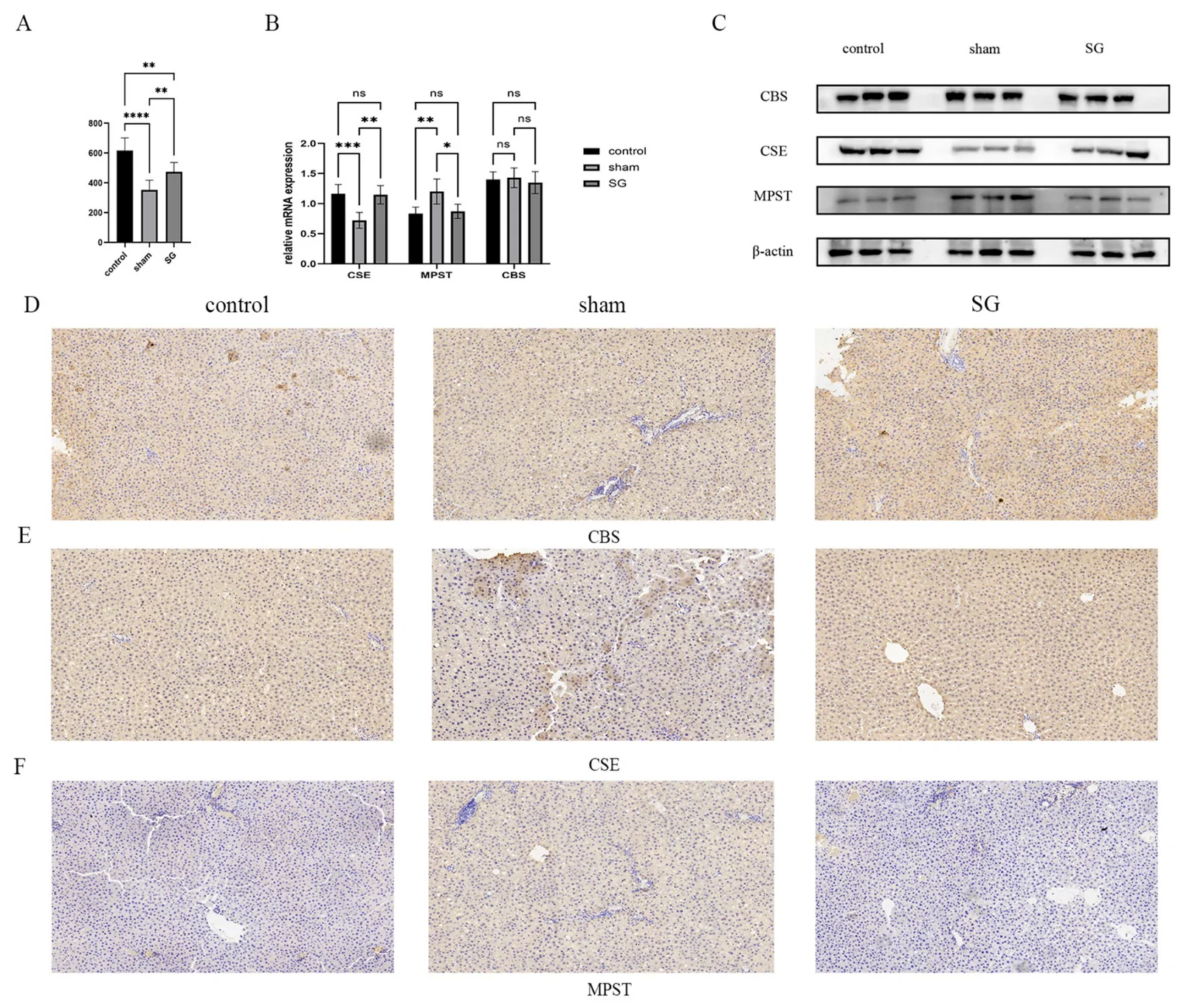
Changes in hepatic H_2_S-related homeostasis following sleeve gastrectomy in HFD-induced obese rats. (**A**) Hepatic H_2_S levels in the Control, Sham, and SG groups. (**B**) Relative mRNA expression of CSE, MPST, and CBS determined by quantitative real-time PCR. (**C**) Representative Western blot images of CBS, CSE, and MPST, with β-actin used as the loading control. (**D**–**F**) Representative immunohistochemical staining of CBS (**D**), CSE (**E**), and MPST (**F**) in liver tissues from the three groups. Compared with the Control group, Sham rats exhibited reduced hepatic H_2_S levels and CSE expression and increased MPST expression, whereas CBS remained relatively unchanged. SG increased hepatic H_2_S and CSE expression and decreased MPST expression compared with the Sham group. Data are presented as mean ± SD. * *p* < 0.05, ** *p* < 0.01, *** *p* < 0.001, and **** *p* < 0.0001; ns, not significant.

The hepatic H_2_S-related sulfide pool was significantly lower in the Sham group than in the Control group. At 8 weeks after surgery, the hepatic H_2_S-related sulfide pool was significantly higher in the SG group than in the Sham group (Figure 5A).

We next examined the expression of the three major H_2_S-producing enzymes. qPCR analysis showed that CSE mRNA expression was reduced in the Sham group compared with the Control group and was markedly increased following SG. Conversely, MPST mRNA expression was increased in the Sham group and significantly reduced after SG. CBS expression remained relatively stable across the three groups, with no marked differences observed (Figure 5B).

Western blot analysis further showed that CSE protein expression was significantly increased in the SG group compared with the Sham group, whereas MPST protein expression was decreased. No significant change in CBS protein expression was observed (Figure 5C). Immunohistochemical staining demonstrated expression patterns consistent with the mRNA and protein analyses (Figure 5D–F).

Overall, the obese rat model showed a pattern of hepatic H_2_S-related alterations broadly similar to that observed in human MASLD samples, characterized by a lower H_2_S-related sulfide pool, lower CSE expression, higher MPST expression, and relatively stable CBS expression. Compared with the Sham group, the SG group exhibited a higher hepatic H_2_S-related sulfide pool and CSE expression, together with lower MPST expression, whereas CBS expression remained relatively unchanged.

## 4. Discussion

This study integrated human liver specimens and a high-fat diet-induced obese rat model to characterize alterations in hepatic H_2_S-related homeostasis in MASLD and changes observed following SG. In patients with MASLD, the hepatic H_2_S-related sulfide pool was lower, accompanied by decreased CSE expression and increased MPST expression, whereas CBS expression remained relatively unchanged. High-fat diet-induced obese rats exhibited a similar pattern. Following SG, improvements in body weight, glucose and lipid metabolism, liver function, and hepatic steatosis were accompanied by an increased hepatic H_2_S-related sulfide pool and CSE expression and decreased MPST expression, while CBS expression remained relatively stable. These findings indicate that MASLD is associated with alterations in hepatic H_2_S-related homeostasis and that changes in this profile accompany metabolic and histological improvements following SG. However, the present findings do not establish whether these H_2_S-related changes contribute causally to the metabolic benefits of SG.

H_2_S is an important endogenous gaseous signaling molecule involved in redox homeostasis, mitochondrial function, inflammation, and glucose and lipid metabolism. CBS, CSE, and MPST are major enzymes involved in endogenous H_2_S generation, although their tissue distribution and functional contributions vary [13]. Importantly, the fluorescence-based signal measured in tissue should not be interpreted as a direct measure of free H_2_S concentration or enzymatic H_2_S production, as it may reflect a detectable H_2_S-related sulfide pool influenced by sulfur metabolism and clearance processes [14]. In the present study, the divergent expression patterns of CBS, CSE, and MPST indicate differential changes in these H_2_S-generating enzymes in association with MASLD and following SG. However, because enzyme activities and H_2_S production fluxes were not directly measured, the functional significance of these expression changes remains uncertain.

A lower hepatic H_2_S-related sulfide pool and decreased CSE expression were observed in both patients with MASLD and obese rats, consistent with previous studies. Peh et al. reported that high-fat feeding reduced hepatic H_2_S biosynthetic capacity and CSE protein expression in mice [8]. Xu et al. further demonstrated suppression of the CSE/H_2_S pathway in human MASLD, high-fat diet-fed mice, and steatotic hepatocytes. Hepatocyte-specific CSE deficiency aggravated hepatic lipid accumulation, insulin resistance, and gluconeogenic abnormalities, whereas H_2_S supplementation partially alleviated these metabolic disturbances [10]. Exogenous H_2_S has also been shown to reduce hepatic triglyceride and cholesterol accumulation and improve oxidative stress in obese mice [9]. These findings support a close association between the CSE/H_2_S system and hepatic lipid homeostasis. However, although the present study identified changes in CSE expression and the hepatic H_2_S-related sulfide pool following SG, it did not determine whether these changes contribute causally to the observed metabolic improvements.

Another notable finding was the increase in MPST expression despite reduced hepatic H_2_S levels. Li et al. reported increased MPST expression in patients with MASLD and high-fat diet-fed mice and showed that free fatty acids could upregulate MPST partly through the NF-κB/p65 pathway. MPST knockdown increased H_2_S levels and attenuated hepatic steatosis, whereas MPST overexpression suppressed H_2_S generation [15]. Mechanistically, MPST interacted with CSE and negatively regulated the CSE/H_2_S pathway, affecting SREBP-1c, JNK signaling, and oxidative stress. Thus, increased MPST expression does not necessarily indicate increased hepatic H_2_S availability. The pattern observed in our study—a lower hepatic H_2_S-related sulfide pool, decreased CSE expression, and increased MPST expression—is broadly consistent with these previous observations. However, because the present study assessed enzyme expression rather than catalytic activity or H_2_S production flux, it remains unclear how these divergent expression changes relate to net hepatic H_2_S production and availability. Following SG, this pattern shifted toward the profile observed in Control animals, with an increased hepatic H_2_S-related sulfide pool and CSE expression and decreased MPST expression.

CBS expression remained largely unchanged in both the human and animal experiments, indicating that MASLD-associated alterations in hepatic H_2_S-related homeostasis were not accompanied by synchronous expression changes in all three major H_2_S-generating enzymes. Unchanged CBS expression should not be interpreted as evidence that CBS is functionally irrelevant to metabolic liver injury. Lambooy et al. demonstrated that liver-specific CBS deletion aggravated hepatic steatosis, inflammation, lipid peroxidation, and liver injury in mice fed a Western diet, supporting a potential protective role of hepatic CBS under metabolic stress [16]. Previous studies have also reported heterogeneous changes in H_2_S-related enzymes across different models [8,15]. Such differences may reflect variations in species, dietary composition, intervention duration, disease severity, and metabolic background. In the present study, CSE and MPST expression showed more consistent changes across human MASLD samples and the rat model, whereas CBS expression remained relatively stable. Because CBS enzymatic activity and sulfur-metabolic flux were not assessed, its functional contribution to MASLD cannot be determined from the present expression data alone.

In our rat model, SG was accompanied by improvements in obesity-related metabolic abnormalities and hepatic histopathology. SG reduced body weight and food intake and improved fasting glucose, liver index, ALT, AST, and most lipid parameters. H&E and Oil Red O staining also demonstrated reduced hepatic steatosis and lipid accumulation. These findings are consistent with clinical evidence showing that bariatric surgery improves steatosis, steatohepatitis, and, in some patients, fibrosis [4]. Lassailly et al. reported resolution of NASH without worsening fibrosis in 84% of patients 5 years after bariatric surgery, with fibrosis regression in 70.2% [17]. The BRAVES trial further showed that bariatric-metabolic surgery was superior to lifestyle intervention plus best medical care in achieving histological resolution of MASH without worsening fibrosis, with 57% of patients in the SG group reaching the primary endpoint at 1 year [18]. Long-term cohort studies have also associated bariatric surgery with reduced risks of major adverse liver and cardiovascular outcomes [19].

Recent clinical evidence supports the beneficial effects of bariatric surgery on liver injury and steatosis. In a prospective study with paired liver biopsies, Pedersen et al. demonstrated that SG significantly reduced the NAFLD Activity Score 12 months after surgery, providing direct histological evidence of improvement in MASLD [20]. Azulai et al. further showed in a large registry-based study that bariatric surgery was associated with reductions in ALT and AST, with changes in liver enzymes not fully explained by the extent of weight loss [21]. These findings, together with other studies reporting improvements in glucose and lipid metabolism and hepatic histology after bariatric surgery [22,23], are consistent with the metabolic, biochemical, and histopathological improvements observed after SG in the present study.

An important unresolved question is whether restoration of H_2_S after SG is a functional mediator of hepatic improvement or a secondary consequence of weight loss and systemic metabolic recovery. SG is a pleiotropic intervention that affects bile acid signaling, gut microbiota, and multiple metabolic pathways in addition to reducing food intake [22,24]. Ryan et al. showed that FXR deficiency attenuated several metabolic benefits of vertical sleeve gastrectomy, whereas Xu et al. demonstrated that CSE-derived H_2_S enhances FXR activity through sulfhydration [22]. These independent findings raise the possibility that SG-induced remodeling of bile acid–FXR signaling may intersect with restoration of the CSE/H_2_S system. However, because bile acid profiles, FXR expression, FXR sulfhydration, and downstream targets were not assessed, this mechanism remains hypothetical.

Importantly, the present findings show an association between metabolic improvement following SG and changes in hepatic H_2_S-related homeostasis but do not establish causality. Although SG was accompanied by an increased hepatic H_2_S-related sulfide pool, increased CSE expression, and decreased MPST expression, no direct intervention targeting these components was performed. Therefore, it remains unclear whether these changes contribute mechanistically to the metabolic benefits of SG or occur secondarily to weight loss, reduced caloric intake, improved insulin sensitivity, or other systemic metabolic adaptations after surgery. This distinction is particularly relevant because SG and caloric restriction may induce distinct hepatic metabolic responses even when comparable weight loss is achieved, as demonstrated by Haran et al. [25]. Moreover, because the present study did not include pair-fed or weight-matched control groups, the effects of SG cannot be fully separated from those of negative energy balance and weight loss. Future studies incorporating genetic or pharmacological manipulation of CSE and MPST, H_2_S donor or inhibitor approaches, and pair-fed or weight-matched controls will be needed to clarify the functional contribution of CSE, MPST, and H_2_S-related signaling to the hepatic effects of SG.

Obesity is also an important potential confounder in the clinical component of this study. BMI was substantially higher in patients with MASLD than in controls; therefore, the observed differences in the hepatic H_2_S-related sulfide pool and CSE and MPST expression cannot be attributed exclusively to MASLD and may partly reflect severe obesity and its associated metabolic milieu. Because obesity, insulin resistance, dyslipidemia, and fatty liver disease are closely interrelated [23], our clinical findings are more appropriately interpreted as showing alterations in hepatic H_2_S-related homeostasis in patients with obesity-associated MASLD. Larger studies with matching or multivariable adjustment for BMI, age, sex, diabetes, and lipid profiles are needed to determine whether these alterations are independently associated with MASLD.

Another limitation of the clinical component is the source of the control liver tissues. Control samples were obtained from non-lesional liver tissue adjacent to hepatic hemangiomas rather than from completely healthy individuals. Although these tissues showed no apparent hepatic steatosis on histological examination and participants with other chronic liver diseases or severe metabolic disorders were excluded, the possibility that the presence of a hepatic hemangioma or its local tissue microenvironment influenced hepatic molecular or metabolic characteristics cannot be completely excluded. These samples should be regarded as non-MASLD surgical controls rather than entirely healthy liver tissue. Future studies using larger and well-characterized control cohorts are warranted to validate the observed alterations in hepatic H_2_S-related homeostasis.

Several methodological considerations regarding H_2_S measurement should also be acknowledged. In the present study, the hepatic H_2_S-related sulfide pool was assessed using a fluorescence-based assay. Because of the dynamic chemical equilibrium among H_2_S and related sulfide species and the characteristics of fluorescence-based detection, the measured signal should be interpreted as reflecting the detectable H_2_S-related sulfide pool under the assay conditions rather than the instantaneous concentration of free H_2_S, the rate of enzymatic H_2_S production, or real-time H_2_S flux [26]. Accordingly, the increased fluorescence signal observed after SG should not be interpreted as direct evidence of enhanced enzymatic H_2_S synthesis. Furthermore, although changes in CSE and MPST expression accompanied alterations in the H_2_S-related sulfide pool, protein and mRNA abundance do not necessarily reflect catalytic activity. Enzymatic activities of CSE, MPST, and CBS were not directly measured in the present study. Future studies combining enzyme activity assays with more specific measurements of H_2_S production and metabolic flux will be required to determine the functional contribution of individual H_2_S-generating enzymes to hepatic sulfur metabolism after SG.

In summary, patients with MASLD and high-fat diet-induced obese rats exhibited altered hepatic H_2_S-related homeostasis, characterized by a reduced H_2_S-related sulfide pool, decreased CSE expression, increased MPST expression, and relatively stable CBS expression. Following SG, improvements in glucose and lipid metabolism, liver function, and hepatic steatosis were accompanied by an increased hepatic H_2_S-related sulfide pool, increased CSE expression, and decreased MPST expression. These findings indicate that changes in hepatic H_2_S-related homeostasis were associated with the metabolic and hepatic improvements observed after SG. However, because CSE and MPST were not directly manipulated and enzymatic H_2_S production was not measured, the functional significance of these changes and their potential causal contribution to the metabolic benefits of SG remain to be determined.

## Figures and Tables

**Table 1:** Primers used in qRT-PCR.

	Genes	Sequence (5′ to 3′)
rat	CBS	F: GAAGGAGAAGTGCCCAGGTTG R: CATTGCTCTTGAACCACCTATCC
	CSE	F: AAATCCGACGAGGAATTGCTT R: GGATGCCACCCTCCTGAAGTAC
	MPST	F: TCTATTCCGCTCCGAGGGTC R: TTCGATGCCATCTCGGGGTT
	β-actin	F: TGCTATGTTGCCCTAGACTTCG R: GTTGGCATAGAGGTCTTTACGG
human	CBS	F: GCGGCTGAAGAACGAAATC R: TAGGTTGTCTGCTCCGTCTGGT
	CSE	F: CAAGTGGCATCTGAATTTGGATT R: GGTTTCTGGTGTAATTGCTGCCT
	MPST	F: CGCCTTCATCAAGACCTACGAG R: GGCTCAGGAAGTCTGTGAAGGG
	β-actin	F: CACCCAGCACAATGAAGATCAAGAT R: CCAGTTTTTAAATCCTGAGTCAAGC

**Table 2:** Baseline clinical and biochemical characteristics of the study participants.

	Control	MASLD	*p* Value
age (year)	41.4 ± 3.7	37.2 ± 2.7	0.387
Sex (male/female)	3/2	2/3	1
Diabetes mellitus, *n* (%)	20%	60%	0.524
height (m)	1.67 ± 0.09	1.71 ± 0.08	0.529
BMI (kg/m^2^)	23.4 (21.3–26.6)	42.3 (37.1–47.6)	0.008
TBil (μmol/L)	18.4 (13.0–23.7)	10.9 (6.4, 15.3)	0.151
DBil (μmol/L)	5.3 (3.3–7.2)	3.1 (1.9–4.3)	0.095
ALB (g/L)	46.4 ± 3.36	42 ± 1.87	0.034
ALTI (U/L)	17.6 ± 6.7	58.6 ± 16.0	0.001
AST (IU/L)	18.8 ± 6.5	40 ± 12.0	0.009
GLU (mmol/L)	6.67 ± 0.86	5.42 ± 0.40	0.019
TC (mmol/L)	4.34 ± 0.35	5.21 ± 0.44	0.009
TG (mmol/L)	1.09 ± 0.28	2.04 ± 0.76	0.033
HDL-C (mmol/L)	1.58 ± 0.16	1.10 ± 0.08	0.002
LDL-C (mmol/L)	1.4 ± 0.13	3.0 ± 0.55	<0.001

## Data Availability

All relevant data from this study are available from the corresponding author.

## Supplementary Materials

The following supporting information can be downloaded at: https://www.mdpi.com/article/10.3390/jcm15218514/s1, File S1: The ARRIVE guidelines 2.0: author checklist.
